# Family Poverty Affects the Rate of Human Infant Brain Growth

**DOI:** 10.1371/journal.pone.0080954

**Published:** 2013-12-11

**Authors:** Jamie L. Hanson, Nicole Hair, Dinggang G. Shen, Feng Shi, John H. Gilmore, Barbara L. Wolfe, Seth D. Pollak

**Affiliations:** 1 Department of Psychology, University of Wisconsin-Madison, Madison, Wisconsin, United States of America; 2 Waisman Center, University of Wisconsin-Madison, Madison, Wisconsin, United States of America; 3 Department of Population Health Sciences, University of Wisconsin-Madison, Madison, Wisconsin, United States of America; 4 Department of Economics, University of Wisconsin-Madison, Madison, Wisconsin, United States of America; 5 Image Display, Enhancement, and Analysis (IDEA) Lab, University of North Carolina at Chapel Hill, Chapel Hill, North Carolina, United States of America; 6 Department of Radiology, University of North Carolina at Chapel Hill, Chapel Hill, North Carolina, United States of America; 7 Biomedical Research Imaging Center (BRIC), University of North Carolina at Chapel Hill, Chapel Hill, North Carolina, United States of America; 8 Department of Psychiatry, University of North Carolina at Chapel Hill, Chapel Hill, North Carolina, United States of America; 9 La Follette School of Public Affairs, University of Wisconsin-Madison, Madison, Wisconsin, United States of America; Hôpital Robert Debré, France

## Abstract

Living in poverty places children at very high risk for problems across a variety of domains, including schooling, behavioral regulation, and health. Aspects of cognitive functioning, such as information processing, may underlie these kinds of problems. How might poverty affect the brain functions underlying these cognitive processes? Here, we address this question by observing and analyzing repeated measures of brain development of young children between five months and four years of age from economically diverse backgrounds (n = 77). In doing so, we have the opportunity to observe changes in brain growth as children begin to experience the effects of poverty. These children underwent MRI scanning, with subjects completing between 1 and 7 scans longitudinally. Two hundred and three MRI scans were divided into different tissue types using a novel image processing algorithm specifically designed to analyze brain data from young infants. Total gray, white, and cerebral (summation of total gray and white matter) volumes were examined along with volumes of the frontal, parietal, temporal, and occipital lobes. Infants from low-income families had lower volumes of gray matter, tissue critical for processing of information and execution of actions. These differences were found for both the frontal and parietal lobes. No differences were detected in white matter, temporal lobe volumes, or occipital lobe volumes. In addition, differences in brain growth were found to vary with socioeconomic status (SES), with children from lower-income households having slower trajectories of growth during infancy and early childhood. Volumetric differences were associated with the emergence of disruptive behavioral problems.

## Introduction

Childhood poverty is a major public health problem. As an estimate of the number of children affected, there are nearly 15 million children in the United States who are currently living in households with incomes below the federal poverty line and another 15 million in other industrialized nations who live in relative poverty [1]. This represents more than 20% of children in the United States and a range across industrialized countries from 4.5% (Iceland) to 25.5% (Romania) While these official rates are staggering, they underestimate the number of children affected by poverty world-wide, as published measurements often rely on outdated assumptions about family expenditures and resources [2], [3].

The full burden of poverty for children frequently includes early and repeated exposure to stress and environmental hazards [for review, see Refs. [4]–[5]. Empirical investigations have noted children living in poverty are exposed to more family turmoil, violence, separation from their families, instability, and receive less social support [6], [7] (as reviewed in Ref. [4]). In addition, children living in poverty generally experience less cognitive stimulation and enrichment in comparison to wealthier children. For example, low-income parents speak less often and in less sophisticated ways to their young children, and are less likely to engage jointly with their children in literary activities such as reading aloud or visiting the library, compared with middle-income parents [8]. Low-income households also tend to have smaller designated play spaces for young children, have fewer home learning resources (e.g., age-appropriate toys, books), and are less likely to have access to a home computer or the Internet [9]. For these reasons, it is not surprising that individuals raised in poor families have elevated rates of learning, behavioral, mental health and physical health problems that persist into adulthood [10]–[13].

Experimental manipulations of income among families, such as conditional cash transfer or welfare-to-work programs are important approaches to study the effect of income on child development, as such programs often increase total income for families at or below the federal poverty line. Economists consider such approaches as one way to study causal effects of SES on child development. Recent work by Duncan, Morris, & Rodrigues [14] pooling nearly 20,000 observations of children ages 2 to 15 found increased household income led to improvements in children's cognitive performance, specifically at younger ages. These investigators, along with Dahl & Lochner [15], found a $1,000 increase in family income raised children's cognitive outcomes, such as math and reading test scores by 6% of a standard deviation. Similar effects have been noted in motivation, social behavior, and externalizing problems with experimental increases in family income leading to better outcomes in these domains [16]–[18]. Such results help to clarify that post-natal environmental experiences do contribute to some of the behavioral differences associated with poverty. Such effects have been found in the United States and also other industrialized countries such as Brazil [19] and Mexico [20], [21]. Preliminary evidence also indicates that such experimental poverty-alleviation programs can positively affect neurobiology, with conditional cash transfer programs in Mexico found to be related to lower salivary cortisol in children [22]. Thus, this body of work moves beyond correlational studies that have shown associations between social class and outcomes and instead provides causal evidence linking increased income in poor families to improvements in outcomes in a number of domains. Some social scientists may see limitations in these data, as these social programs are not specifically designed to affect outcomes for children therefore the exact mechanisms of any observed change are unclear [23]. It is not known what lies behind the positive response to increased income or the negative effect of poverty on children. We still know very little about how impoverished environments lead to developmental problems.

Studies in non-human animals where environmental conditions can be experimentally manipulated suggest candidate mechanisms for how environmental experience might affect central nervous system functioning. Conditions such as the variety and complexity of the stimuli in an animal's cage can influence different aspects of brain structure, including the number of neurons, glial cells, myelination, dendrites, synapses, and neurogenesis (for review, see Ref. [23]–[24]). Such environmental variables capture some aspects of extremely low-income home environments. Animals living in environments with lower amounts of cognitive stimulation or greater amounts of stress tend to have smaller brains and fewer cell bodies, dendrites and synapses than animals reared in more normative environments [25]–[27]. Remodeling of these cellular components (e.g., neural cell bodies, dendrites and synapses) are theorized to underlie changes in gray matter, one type of brain tissue [27].

The development of gray matter is especially important for understanding problems in cognition and behavioral regulation because this brain tissue contains neural cell bodies, dendrites, synapses that support the processing of information and execution of actions. A large body of research has focused on these types of changes because learning is believed to be related to this kind of neural reorganization [27], [28]. Developmental cognitive neuroscience research in humans has found gray matter and also white matter, the other major type of tissue in the brain, are vulnerable to environmental perturbations [29], [30]. White matter is composed of myelinated fiber tracts and aids in helping distal portions of the brain work together. Important research in human twins examining genetic and environmental contributions of brain development however finds gray matter may be uniquely affected by the early environment and is less heritable than white matter [31].

Research focused specifically on the neurocognitive effects of poverty helps to further clarify possible changes in the brain, with recent studies providing evidence of SES influences on executive function (for review, see Ref. [32]). The frontal lobe has been implicated in executive functions such as planning, impulse control, and control of attention, making it a candidate structure for investigation [33]. This brain region also has a protracted course of post-natal development and may be particularly vulnerable to the effects of early stress and experience [34]. Additionally, alterations in the frontal lobe may be particularly important for the elevated rates of learning, behavioral, and health problems seen in children from low SES backgrounds. Exposure to poverty has been associated with decrements in attentional processes, working memory, and inhibitory control during infancy [35], [36], childhood and adolescence [37]–[39], and also in adulthood [40], [41]. The results of longitudinal research suggest that increased duration of a child's exposure to poverty is related to greater deficits in executive function and working memory in adulthood [42]. The nascent body of research employing measures of neurobiology such as electroencephalography (EEG) or MRI also point to possible alterations in the frontal lobe being associated with poverty. In young children [43], [44] and adolescents [45], differences have been found in the resting frontal EEG, with lower activity being noted for children living in poverty. Otero [42] suggested this result reflected a maturational lag in frontal lobe development. Further work examining SES, behavioral performance, and the neural correlates of selective attention has found differences in evoked brain activity. Young children from lower-SES backgrounds display lower electrical activity when deploying different aspects of selective attention, a cognitive process dependent on the frontal lobe [46]–[48].

Based upon these ideas, we examined changes in human brain structure from birth through the toddler years. Participants in this study ranged from 5 months to 4 years of age, covering a period during which there is a great deal of post-natal brain growth. Indeed, gray matter development accounts for most of the human brain's growth during the first few years of post-natal life [49]. We predicted that children from Low SES, as indexed by lower household income, would have lower volumes in total gray matter and frontal lobe gray matter. We hypothesized that these differences would not be present early in development and would increase over time. Given the importance of the frontal lobe in behavioral regulation [50], we also hypothesized that variations in this brain area (both in regards to lower volume and slower growth) would be related to greater disruptive behavioral problems in children (as measured by the Child Behavior Checklist, CBCL). In order to test this idea, we examined whether growth trajectories for these brain regions were altered as toddlers were increasingly exposed to impoverished environments.

We analyzed two hundred and three MRI scans from seventy-seven infants living in lower SES households and also those living in more affluent households to test our hypothesis. Most of the infants were followed longitudinally, with the average first scan at age 13 months and subsequent scans approximately every half-year until children were four years old. We employed a method that provides an extremely sensitive way of detecting changes in infant brain growth. The infants in this study represent families with annual incomes that ranged from extreme poverty (less than $5,000 a year in income) to over $100,000 annually.

## Methods

### Subjects and Recruitment Information

Data were derived from the US National Institute of Health MRI Study of Normal Brain Development. This is a multi-site, longitudinal study of typically developing children, from ages newborn through young adulthood, conducted by the Brain Development Cooperative Group and supported by the National Institute of Child Health and Human Development, the National Institute on Drug Abuse, the National Institute of Mental Health, and the National Institute of Neurological Disorders and Stroke (Contract #s N01-HD02-3343, N01-MH9-0002, and N01-NS-9-2314, -2315, -2316, -2317, -2319 and -2320). A listing of the participating sites and a complete listing of the study investigators can be found at www.bic.mni.mcgill.ca/nihpd/info/participating_centers.html.

Participants were recruited from the greater Boston and Saint Louis metropolitan regions via a community- based strategy that included hospital venues (e.g., maternity wards and nurseries, satellite physician offices, and well-child clinics), community organizations (e.g., day- care centers, schools, churches, and other types of community centers), and siblings of children participating in other studies (details provided in Ref. [51]). Participants were excluded based on demographic (e.g., child adopted; medical history unknown), pregnancy (e.g., intrauterine exposure to teratogens such as cigarette smoke or alcohol; use of general anesthesia during childbirth), delivery (e.g., C-section with fetal or maternal distress; high forceps or vacuum extraction), other birth-neonatal complications (e.g., anemia; respiratory distress; hospital admission for specialized care), child development (e.g., significant language/learning disorder; lead treatment; muscle disease; maternal medications during breastfeeding; child head injury), and family psychiatric history criteria. For full discussion of these criteria, see Ref. [51].

The initial sample consisted of 110 healthy children (newborn through 4-years 5-months of age), demographically- balanced to mirror proportions defined by the United States Census Bureau in terms of gender, race, ethnicity, and income distribution. A total of 338 MRI scans were acquired from these participants over time. We were able to segment scans from two hundred and three MRI images (representing seventy-seven infants). Fifty-five infants were followed longitudinally (Average age at first scan = 13.5 +/−7.9 months; Average number of scans  = 3.1; Average amount of time between scans = 6.5+/−4.1 months) and an additional twenty-two infants were scanned once at various ages (Average age = 17.9+/−11.9 months). Participants were drawn from families with incomes ranging from barely 4% to over 400% of the federal poverty level (FPL). Demographics of the sample are noted in Table 1. Data on children's behavioral and emotional problems were collected using the Child Behavior Checklist (CBCL) [52].

**Table 1:** Subject Demographics.

	Total Subjects (n = 77)	Total Scan (n = 203)
Gender (Male)	46	115
Maternal Education		
Less Than High School	1	1
High School	72	187
Some College	1	1
College Degree	1	2
Greater than a College Degree	2	5
Education Information Missing	0	7
Household Income		
$0–5000	2	5
$5001–10000	2	4
$10001–15000	2	6
$15000–25000	2	9
$25001–35000	7	19
$35001–50000	12	25
$50001–75000	21	68
$75001–100000	11	27
Greater than $100001	18	32
Sample characteristics in relation to federal poverty line (FPL)		
Below 200% FPL	16	45
Between 200–400% FPL	32	102

### Ethics Statement

Written informed consent from the parents/guardians of all children was obtained in compliance with research standards for human research at Children's Hospital Boston and Washington University in St. Louis. All procedures were in accordance with the Helsinki Declaration. The Institutional Review Board at the University of Wisconsin-Madison and University of North Carolina also approved the analysis of this human subjects data.

### MRI Acquisition and Processing

T1- and T2-weighted scans were obtained with a GE (General Electric, Milwaukee, WI) or Siemens (Siemens AG, Erlangen, Germany) MRI scanner. A 2D multi-slice spin-echo T1 sequence was used with- TR: 500 ms, TE: 10 ms for GE, 12 ms for Siemens, and 3 mm slice thickness. T2 images were acquired via a 2D multi-slice dual-echo fast/turbo spin echo with the following parameters- TR: 3500 ms, TE1: 17 ms, TE2: 119 ms, and 3 mm slice thickness. Both scans provided coverage from the apex of head to bottom of the cerebellum.

**Figure 1:**
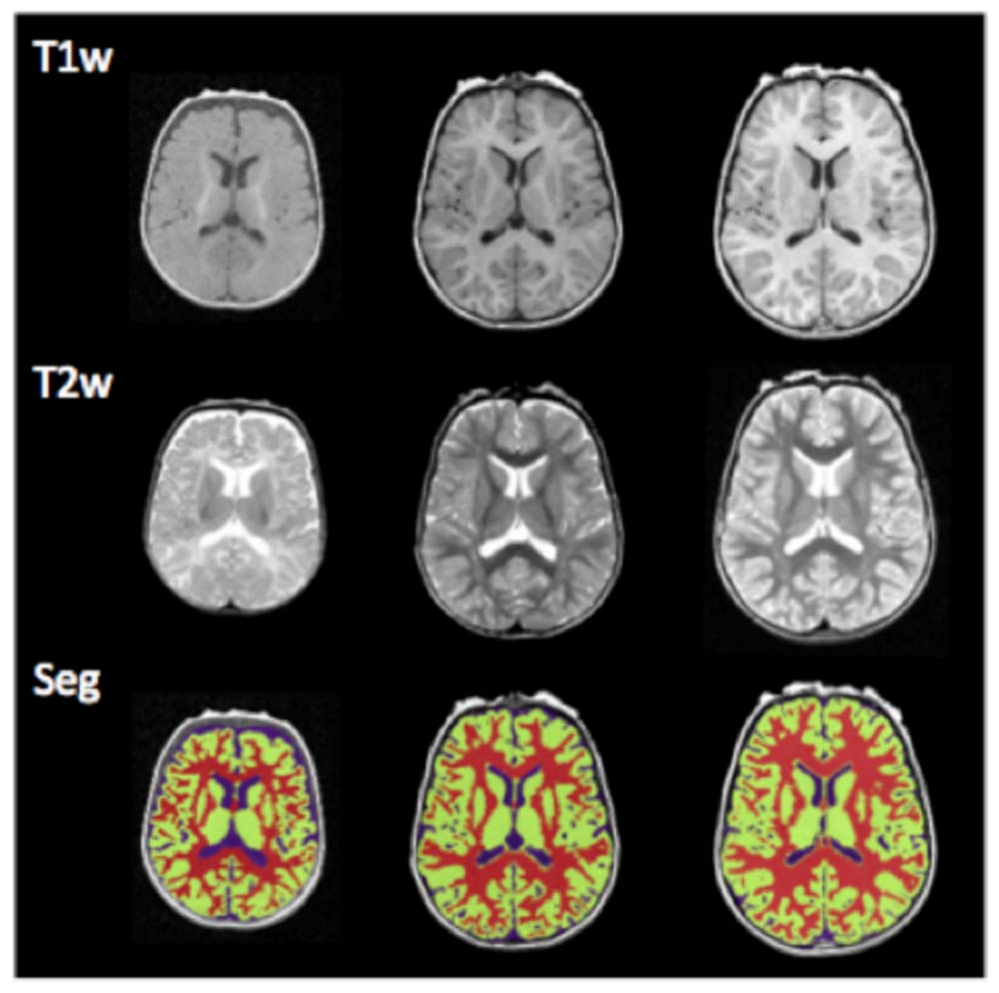
This figure shows example axial slices from a typical subject scanned at birth (left column), 2 (middle column), and 4 years old (right column). T1 MRI, T2 MRI, and segmented gray matter (green) and white matter (red) are provided.

All structural scans had non-brain tissue (e.g., skull and dura) automatically removed and were then bias corrected with nonparametric non-uniform intensity normalization methods to reduce the impact of intensity inhomogeneity [53]–[55]. All images were segmented via an Expectation–Maximization (EM) algorithm [56] with infant brain atlases representing subject-independent population information (as detailed in Ref. [57]; infant brain templates are available for download at http://www.nitrc.org/projects/pediatricatlas). This segmentation involved two iterative steps: 1) a registration step for aligning an age-specific atlas onto a given image and 2) a segmentation step for estimating brain tissues using the MRI intensity distribution from the image in conjunction with the aligned tissue probability maps from atlas. For participants with longitudinal data, segments of the last time-point image were alternatively employed as subject-specific atlas for guiding segmentations of early time-point images for better accuracy [58]. These procedures aided in robustly registering and identifying tissue structures across age groups and subjects. A brain atlas labeled with gray matter, white matter, and the four primary lobes of the brain (i.e., frontal, temporal, parietal, occipital) was employed to label the whole-brain [58]. This atlas was originally defined on the Montreal Neurological Institute (MNI) single subject brain MR image and was later adapted for infant neuroimages [57], [59]. For each subject, total gray, white, and cerebral (summation of total gray and white matter) volumes were calculated, along with gray matter volume of the frontal, parietal, temporal, and occipital lobes. Example structural scans and resulting segments are shown in Figure 1.

Imaging data was examined pre- and post-processing to improve the reliability of segmentation results. First, as a screening step, images were labeled by an expert in infant neuroimaging (FS) as “pass” or “fail” corresponding to whether image quality (e.g., motion, contrast) was sufficient or not to separate brain tissues. For example, white matter varies in contrast for cortical versus subcortical tissue. MRI volumes with poor image quality do not allow for robust segmentation of these different types of white matter. Elimination due to image quality accounted for 80% of discarded scans. Second, as an evaluation step, segmented images were reviewed one by one by the rater and those with visible skull-stripping or segmentation errors were discarded. Segmentation failure rates were similar to those previously reported in the infant imaging literature [49]. Discarded scans did not differ by group (Pearson *χ* ^2^ p = .233).

### Statistical Analyses

Random effects models were constructed to assess differences in average brain volumes by family economic status controlling for participant birth weight, sex, and age in months (quadratic polynomial). Birth weight serves as an indicator of both an infant's early health and individual differences in head/brain size. The quadratic polynomial for the effects of age allow for a concave or convex growth pattern rather than imposing an assumption of linear age effects. An indicator of SES was constructed using reported household income. If a subject had multiple study visits, household income was averaged over visits to create a measure of permanent income (since annual inflation over the 2001–2007 period was relatively low and stable, averaging 2.7 percent).

Families were divided into three groups relative to the federal poverty level (FPL): (i) low SES families with household income at or below 200 percent of the FPL, (ii) moderate SES families with household income between 200 and 400 percent of the FPL, and (iii) high SES families with household income above 400 percent of the FPL. These categories have been used in previous work on disparity and inequality within the social science, public policy, and health literatures, along with being employed by the United States Department of Health and Human Services (e.g., [60]). For this analysis, brain volumes of interest included: total gray, white and cerebral (summation of total gray and white matter) volumes, along with the gray matter volumes of the frontal, parietal, temporal, and occipital lobes. The random effects model for the *i*th subject at time *t* took the form-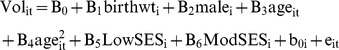



Mixed-effects linear models (growth models) were constructed to extend our analysis to examine differences in brain volume trajectories. This approach is consistent with work on overall health, which finds that lower income during childhood is associated with poorer overall health and higher instances of health problems and, moreover, that this gradient steepens with a child's age [61]. Covariates for the growth models included birth weight and sex. The mixed-effects linear model for the *i*th subject at time *t* took the form-
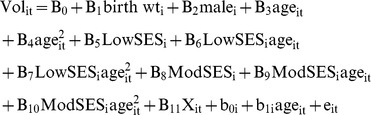



The terms b_0i_ and b_1i_ are the subject-specific random intercept and random slope. The growth model analysis focused on volumes that were observed to be affected, on average, by low SES.

Finally, we explored whether observed delays in infant brain development may be associated with aspects of children's behavior. Random effects models were constructed to examine the influence of brain development (both absolute brain volumes and growth rates) on children's maladaptive behavioral and emotional problems as measured by the Child Behavior Checklist (CBCL). The analysis focused specifically on children's internalizing (i.e., anxious, depressive) and externalizing (i.e., aggressive, hyperactive) behaviors. Covariates included participant birth weight, sex, and age in months.

## Results

The sample of infants was representative of the population of children living in poverty in the United States. Among minorities Black/Non Hispanic and Hispanic/Black, we replicate demographics in the US (two groups combined- 45.5% in our sample and 40% nationally). Nationally, nineteen percent of the population is living below the poverty line; in our sample it is 20% of infants. We have more two-parent families in the study than nationally and low-birth weight of children was used as an exclusionary criterion during recruitment. This makes our estimates of group differences conservative and likely underestimates the effects of poverty on children's brain development.

As hypothesized, when compared to children from high SES families, children from poor and near poor households (family incomes below 200% FPL) were found to have significantly lower average total gray matter volumes (β = −32,345.3, p = 0.021). This represents a difference of 0.40 standard deviations compared to the overall sample average for total gray matter volume (568,837±80,812). We also examined whether there are specific regions of the infant brain that are particularly sensitive to the effects of early poverty. Children from poor and near poor households were additionally found to have significantly lower average frontal (β = −10,983.1, p = 0.020) and parietal (β = −6,290.1, p = 0.017) gray matter volumes. These differences are large, representing deficits of 0.47 and 0.40 standard deviations, respectively. The estimated differences in total, frontal and parietal gray matter volumes among children in the low SES group remain statistically significant after adjusting for multiple comparisons according to the Benjamini-Hochberg (False Discovery Rate) procedure. All results also remain the same if scanner type is added as a covariate of no interest.

We did not detect statistically significant differences in total cerebral volume (summation of total gray and white matter) or white matter volumes. In addition, no differences were found for regional gray matter volumes of the parietal or temporal lobes. The association between family economic status and average brain volumes was found to be concentrated among the most impoverished children. We did not detect statistically significant differences in any brain volumes of interest when comparing children from moderate SES households to children from high SES families.

**Figure 2:**
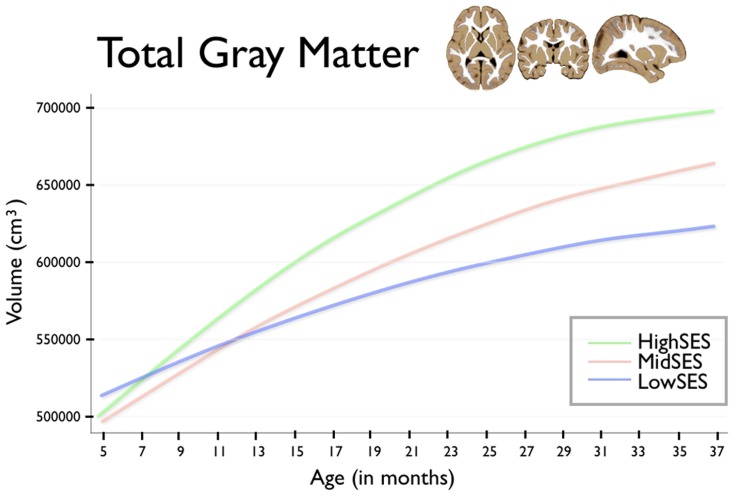
This figure shows total gray matter volume for group by age. Age in months is shown on the horizontal axis, spanning from 5 to 37 months. Total gray matter volume is shown on the vertical axis. The blue line shows children from Low SES households; children from Mid SES households are shown in red. The green line shows children from High SES households.

**Figure 3:**
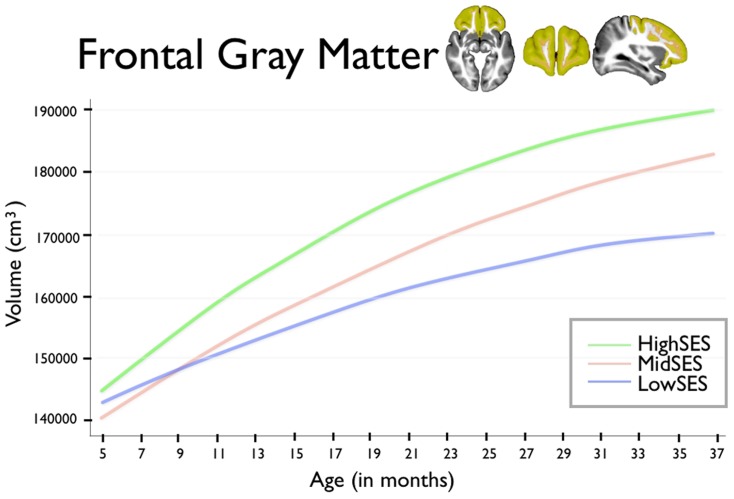
This figure shows frontal lobe gray matter volumes for group by age. Age in months is shown on the horizontal axis, spanning from 5 to 37 months. Total gray matter volume is shown on the vertical axis. The blue line shows children from Low SES households; children from Mid SES households are shown in red. The green line shows children from High SES households.

**Figure 4:**
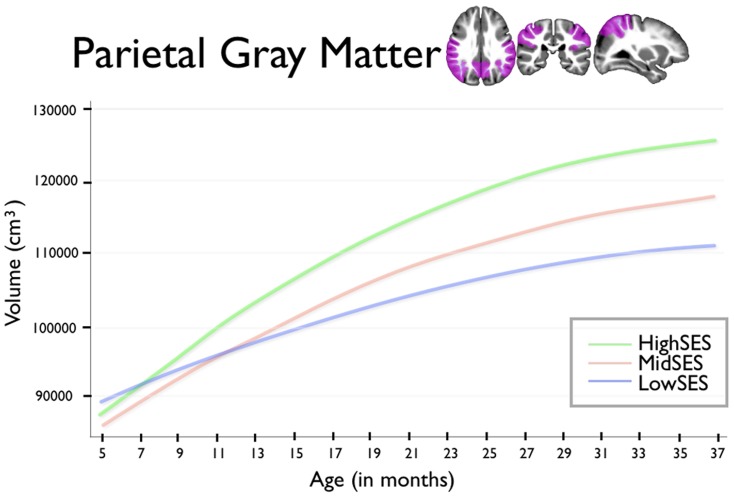
This figure shows parietal lobe gray matter volumes for group by age. Age in months is shown on the horizontal axis, spanning from 5 to 37 months. Total gray matter volume is shown on the vertical axis. The blue line shows children from Low SES households; children from Mid SES households are shown in red. The green line shows children from High SES households.

For brain volumes where we found strong associations with early poverty – total gray matter volume and the gray matter volumes of the frontal and parietal lobes – we examined whether these associations with family economic status extended to growth trajectories. We see children from low SES households showed reduced total gray matter growth trajectory (age β = −6,599.7, p<0.001; age∧2 β = 91.5, p = 0.004) when compared to that of high SES children. Similar patterns of reduced growth trajectory in children from low SES households were also found for the frontal lobe trajectory (age β = −1,234.2, p = 0.019; age∧2 β = 16.4, p = 0.1) and parietal lobe trajectory (age β = −1,187.1, p = 0.003; agê2 β = 16.3, p = 0.036). For children from low, moderate, and high SES families, growth trajectory for total gray matter is presented in Figure 2, for frontal lobe gray matter in Figure 3, and for parietal lobe gray matter in Figure 4.

Finally, we explored the potential implications of the differences we observed in infant brain development for aspects of children's behavior by examining internalizing and externalizing symptomology on the CBCL. Most subjects were within the normative range for both scales (>63 being in the clinical range); for externalizing, the 90^th^ percentile of the data was a score of 56. We found that lower total gray matter was associated with greater externalizing symptoms such as rule breaking, excessive aggression, and hyperactivity in the children by age 4 years (β = −0.0000394, *p* = .05). In addition, there was an association between externalizing symptoms and frontal lobe gray matter (β = −0.000185, *p* = .004), while no such association was seen for the parietal lobe gray matter (β = −0.0000549, *p* = .584). Such behavior problems in young children are risk factors for increasingly serious and persistent mental health issues in adulthood [52]. Looking at whether absolute volumetric variations or differences in growth rate were related to externalizing symptoms, total gray matter volume (as opposed to growth) was more strongly related to these problem behaviors (volume β = −0.0000409, *p* = .080; growth β = −0.000227, *p* = .358). For the frontal lobe, we observe a statistically significant association with growth and volume, with initial evidence suggesting volume, rather than growth, has a stronger influence on externalizing symptoms for low SES children (volume β = −0.000158, *p* = .049; growth β = −0.00138, *p* = .045). There was no association between internalizing symptomology and total gray matter (p = 0.366), frontal lobe gray matter volume (p = 0.496), or parietal lobe gray matter (p = 0.312). SES was not associated with externalizing symptoms in this sample; selection of the sample and numerous exclusionary criteria likely explain why we do not find this expected link.

## Discussion

These unique data suggest that low SES environments influence the rate of human infant brain development. Infants, toddlers and preschoolers from lower income families began their lives with similar gray matter brain volumes but had lower total gray matter compared with those from middle and high-income households by toddlerhood. Differences in brain volumes between children from low and high SES households are not accounted for by infant birth weight, infant's early health, or differences in head size at birth. As infants aged—and presumably had increased exposure to the effects of their environments— the differences in brain volume between poor children and those with greater resources widened. Smaller volumes in this brain tissue were related to greater behavior problems in the pre-school years.

The differences seen were localized to the frontal and parietal volumes, with children from lower income families having smaller volumes in these brain regions. A large body of research has found the frontal lobe is centrally involved with executive functions such as planning, impulse control, and control of attention [33]. Such differences fit well with previous research noting poorer executive function in in children from lower SES backgrounds [62]. The parietal lobe is important for sensory integration and aspects of visual attention [63]. Development of the parietal lobe may be particularly important for connectivity between brain regions [64].

These results extend a consistent literature in rodents, non-human primates, and humans suggesting that early environments marked by stress or deprivation negatively influence brain development [65]–[69]. This emerging body of research has found differences in brain structure in portions of the frontal lobe, which fits well with the analysis presented here [68]. These findings suggest that aspects of low SES environments have important functional implications for children's health and adaptation [70], perhaps by influencing key features of central nervous system development. In regards to neurobiological mechanisms, the differences in volume we find are likely due to neuronal remodeling, rather than birth of new neurons (or neurogenesis) [27], [71], [72]. Volumetric differences associated with environmental experience are likely related to an increase in synapses along with increases in supportive tissue, including both capillaries and glia [27]. Studies with mice find changes in the hippocampus as measured by MRI to correlate strongly with axonal growth markers and not with measures of neuronal size or number, again pointing to remodeling of neuronal processes rather than neurogenesis. Further work is however needed to fully understand such changes as alterations in neurogenesis, synaptogenesis and neuronal morphology could all be driving volumetric changes (for review, see Ref. [72]). In future research, we also aim to employ higher resolution MRI methods in order to more precisely quantify areas implicated by previous research such as the hippocampus, specific portions of the frontal lobe, or smaller brain structures involves with language functions (e.g., Broca's and Wernicke's areas). Additional use of novel MRI methods, such as diffusion tensor imaging would also be beneficial, as initial investigations have found aspects of white matter integrity are related to SES [73].

This sample was economically diverse: children came from families with incomes significantly below the federal poverty level (FPL) as well as from families with incomes over 400% of the FPL. Specifically, children in this study were drawn from families with incomes ranging from barely 4% to over 400% of the FPL. Yet, these data cannot address issues of causation. This is because poverty carries multiple components of environmental risk. Other “third” variables, not measured in our sample, could lead to or be related to both lower family SES and differential rates of infant brain growth. Future research will be necessary to determine if one critical aspect of the environment is likely to influence children's brain development, or whether such effects reflect the influence of multiple factors in combination. Candidate factors might include the effects of household resources, environmental stimulation, crowding, exposure to pathogens and noise, parental stress, and nutrition. It is also possible that pre-natal experiences affect brain development and reflect other disadvantages and risks related to poverty. Because humans are able to adapt to a range of environmental conditions, we must understand more about the level at which impoverished environments become toxic for children.

Of important note, this data set was designed to study normative development and screened out infants based on demographic, birth-neonatal complications, child development, and family psychiatric history criteria (as noted in our method section, also see Ref. [41]). This design may skew the sample because such issues are disproportionately represented among impoverished children. The present results therefore reflect so-called “normal” children living in lower SES. Our results likely under-represent the true effects of SES. Alternatively one could argue that the exclusionary criteria may strengthen the implications of our results as such factors as possible explanations of the association can largely be ruled out as factors lying behind the findings reported.

Increased understanding about how environmental variations, such as socio-economic disparities, affect human brain development and behavior has significant implications for advancing basic scientific questions such as understanding genetic versus environmental contributions of brain and behavioral development. But even more important is that such understanding should lead to public policy initiatives directed at improving and decreasing disparities in human capital.

## References

[1] United Nations Children's Fund: UNICEF (2012) Progress for Children: A report card on adolescents, No. 10. Available: http://www.unicef.org/publications/index_62280.html. Accessed 20 December 2011.

[2] Betson DM, Citro CF, Michael RT (2000) Recent developments for poverty measurement in US official statistics. Journal of Official Statistics 16: 87–112.

[3] Gershoff ET, Aber JL, Raver CC, Lennon MC (2007) Income Is Not Enough: Incorporating Material Hardship Into Models of Income Associations With Parenting and Child Development. Child Dev 78: 70–95. 17328694 10.1111/j.1467-8624.2007.00986.x PMC2835994

[4] Evans GW (2004) The environment of childhood poverty. American Psychologist 59: 77–92. 14992634 10.1037/0003-066X.59.2.77

[5] Bradley RH, Corwyn RF (2002) Socioeconomic status and child development. Annual Review of Psychology 53: 371–399. 10.1146/annurev.psych.53.100901.135233 11752490

[6] Atkinson T, Liem R, Liem JH (1986) The social costs of unemployment: Implications for social support. Journal of Health and Social Behavior 27: 317–331. 3559126

[7] Sampson RJ, Raudenbush SW, Earls F (1997) Neighborhoods and violent crime: A multilevel study of collective efficacy. Science 277: 918–924. 9252316 10.1126/science.277.5328.918

[8] Hart B, Risley TR (1995) Meaningful Differences in the Everyday Experience of Young American Children. Baltimore: Paul Brookes. 268 p.

[9] Newsom J, Newsome E (1976) Seven years old in the home environment. Oxford, England: John Wiley & Sons. 436 p.

[10] Barajas RJ, Philipsen N, Brooks-Gunn J (2007) Cognitive and emotional outcomes for children in poverty. In: Crane DR, Heaton TB, editors. Handbook of Families and Poverty. Thousand Oaks, CA: Sage. pp. 311–333.

[11] Evans GW, Kim P (2010) Multiple risk exposure as a potential explanatory mechanism for the socioeconomic status-health gradient. Ann N Y Acad Sci 1186: 174–189. 20201873 10.1111/j.1749-6632.2009.05336.x

[12] Duncan G, Yeung W, Brooks-Gunn J, Smith J (1998) How much does childhood poverty affect the life chances of children? American Sociological Review 63: 406–423.

[13] Haveman R, Wolfe B (1995) The determinants of children's attainments: A review of methods and findings. Journal of Economic Literature 33: 1829–1878.

[14] Duncan GJ, Morris PA, Rodrigues C (2011) Does money really matter? Estimating impacts of family income on young children's achievement with data from random-assignment experiments. Developmental Psychology 47: 1263–1279. 21688900 10.1037/a0023875 PMC3208322

[15] Dahl GB, Lochner L (2012) The impact of family income on child achievement: Evidence from the earned income tax credit. The American Economic Review 102: 1927–1956.

[16] Duncan GJ, Huston AC, Weisner TS (2007) Higher ground: New hope for the working poor and their children. New York, NY: Russell Sage Foundation. 172 p.

[17] Huston AC, Duncan GJ, Granger R, Bos J, McLoyd V, et al (2001) Work-based antipoverty programs for parents can enhance the school performance and social behavior of children. Child Dev 72: 318–336. 11280487 10.1111/1467-8624.00281

[18] Huston AC, Duncan GJ, McLoyd VC, Crosby DA, Ripke MN, et al (2005) Impacts on children of a policy to promote employment and reduce poverty for low-income parents: New hope after 5 years. Developmental Psychology 41: 902–918. 16351336 10.1037/0012-1649.41.6.902

[19] Glewwe P, Kassouf AL (2012) The impact of the Bolsa Escola/Familia conditional cash transfer program on enrollment, dropout rates and grade promotion in Brazil. Journal of Development Economics 97: 505–517.

[20] Fernald LC, Gertler PJ, Neufeld LM (2008) Role of cash in conditional cash transfer programmes for child health, growth, and development: an analysis of Mexico's Oportunidades. The Lancet 371: 828–837. 10.1016/S0140-6736(08)60382-7 PMC2779574 18328930

[21] Gertler P (2004) Do conditional cash transfers improve child health? Evidence from PROGRESA's control randomized experiment. The American Economic Review 94: 336–341. 29068185 10.1257/0002828041302109

[22] Fernald LC, Gunnar MR (2009) Poverty-alleviation program participation and salivary cortisol in very low-income children. Social Science & Medicine 68: 2180–2189. 19406546 10.1016/j.socscimed.2009.03.032 PMC2768580

[23] Leroy JL, Ruel M, Verhofstadt E (2009) The impact of conditional cash transfer programmes on child nutrition: a review of evidence using a programme theory framework. Journal of Development Effectiveness 1: 103–129.

[24] Grossman AW, Churchill JD, McKinney BC, Kodish IM, Otte SL, et al (2003) Experience effects on brain development: possible contributions to psychopathology. Journal of Child Psychology and Psychiatry 44: 33–63. 12553412 10.1111/1469-7610.t01-1-00102

[25] Rosenzweig MR, Krech D, Bennett EL, Diamond MC (1962) Effects of environmental complexity and training on brain chemistry and anatomy: a replication and extension. Journal of Comparative and Physiological Psychology 55: 429–437. 14494091 10.1037/h0041137

[26] Ferchmin PA, Eterovic VA, Caputto R (1970) Studies of brain weight and RNA content after short periods of exposure to environmental complexity. Brain Research 20: 49–57. 5444768 10.1016/0006-8993(70)90153-8

[27] Anderson BJ (2011) Plasticity of gray matter volume: The cellular and synaptic plasticity that underlies volumetric change. Dev Psychobiol 53: 456–465. 21678393 10.1002/dev.20563

[28] Greenough WT, Bailey CH (1988) The anatomy of a memory: Convergence of results across a diversity of tests. Trends in Neurosciences 11: 142–147.

[29] Lupien SJ, McEwen BS, Gunnar MR, Heim C (2009) Effects of stress throughout the lifespan on the brain, behaviour and cognition. Nat Rev Neurosci 10: 434–445. 19401723 10.1038/nrn2639

[30] Teicher MH, Tomoda A, Andersen SL (2006) Neurobiological consequences of early stress and childhood maltreatment: are results from human and animal studies comparable? Ann N Y Acad Sci 1071: 313–323. 16891580 10.1196/annals.1364.024

[31] Gilmore JH, Schmitt JE, Knickmeyer RC, Smith JK, Lin W, et al (2010) Genetic and environmental contributions to neonatal brain structure: a twin study. Human Brain Mapping 31: 1174–1182. 20063301 10.1002/hbm.20926 PMC3109622

[32] Hanson JL, Hackman DA (2012)Cognitive neuroscience and SES disparities. In: Wolfe BL, Evans WN, Seeman TE, editors. Biological Consequences of Socioeconomic Inequalities, New York, NY : Russell Sage Foundation. 292 p.

[33] Stuss DT (2011) Functions of the frontal lobes: relation to executive functions. Journal of the International Neuropsychological Society 17: 759–765. 21729406 10.1017/S1355617711000695

[34] Toga AW, Thompson PM, Sowell ER (2006) Mapping brain maturation. Trends Neurosci 29: : 148–159. 10.1016/j.tins.2006.01.007 PMC3113697 16472876

[35] Lipina SJ, Martelli MI, Colombo JA (2005) Performance on the A-not-B task of Argentinean infants from unsatisfied and satisfied basic needs homes. Revista interamericana de psicología 39: 49–60.

[36] Mezzacappa E (2004) Alerting, orienting, and executive attention: Developmental properties and sociodemographic correlates in an epidemiological sample of young, urban children. Child Dev 75: 1373–1386. 15369520 10.1111/j.1467-8624.2004.00746.x

[37] Farah MJ, Shera DM, Savage JH, Betancourt L, Giannetta JM, et al (2006) Childhood poverty: Specific associations with neurocognitive development. Brain research 1110: 166–174. 16879809 10.1016/j.brainres.2006.06.072

[38] Noble KG, McCandliss BD, Farah MJ (2007) Socioeconomic gradients predict individual differences in neurocognitive abilities. Developmental Science 10: 464–480. 17552936 10.1111/j.1467-7687.2007.00600.x

[39] Noble KG, Norman MF, Farah MJ (2005) Neurocognitive correlates of socioeconomic status in kindergarten children. Developmental Science 8: 74–87. 15647068 10.1111/j.1467-7687.2005.00394.x

[40] Singh-Manoux A, Richards M, Marmot M (2005) Socioeconomic position across the lifecourse: how does it relate to cognitive function in mid-life? Annals of epidemiology 15: 572–578. 16118001 10.1016/j.annepidem.2004.10.007

[41] Turrell G, Lynch JW, Kaplan GA, Everson SA, Helkala EL, et al (2002) Socioeconomic position across the lifecourse and cognitive function in late middle age. The Journals of Gerontology Series B: Psychological Sciences and Social Sciences 57: S43–S51. 10.1093/geronb/57.1.s43 11773232

[42] Evans GW, Schamberg MA (2009) Childhood poverty, chronic stress, and adult working memory. Proc Natl Acad Sci U S A 106: 6545–6549. 19332779 10.1073/pnas.0811910106 PMC2662958

[43] Otero GA (1997) Poverty, cultural disadvantage and brain development: a study of pre-school children in Mexico. Electroencephalography and clinical neurophysiology 102: 512–516. 9216484 10.1016/s0013-4694(97)95213-9

[44] Otero GA, Pliego-Rivero FB, Fernández T, Ricardo J (2003) EEG development in children with sociocultural disadvantages: a follow-up study. Clinical neurophysiology 114: 1918–1925. 14499754 10.1016/s1388-2457(03)00173-1

[45] Tomarken AJ, Dichter GS, Garber J, Simien C (2004) Resting frontal brain activity: linkages to maternal depression and socio-economic status among adolescents. Biological Psychology 67: 77–102. 15130526 10.1016/j.biopsycho.2004.03.011

[46] D'Angiulli A, Herdman A, Stapells D, Hertzman C (2008) Children's event-related potentials of auditory selective attention vary with their socioeconomic status. Neuropsychology 22: 293–300. 18444707 10.1037/0894-4105.22.3.293

[47] Stevens C, Lauinger B, Neville H (2009) Differences in the neural mechanisms of selective attention in children from different socioeconomic backgrounds: an event-related brain potential study. Developmental science 12: 634–646. 19635089 10.1111/j.1467-7687.2009.00807.x PMC2718768

[48] Kishiyama MM, Boyce WT, Jimenez AM, Perry LM, Knight RT (2009) Socioeconomic disparities affect prefrontal function in children. Journal of cognitive neuroscience 21: 1106–1115. 18752394 10.1162/jocn.2009.21101

[49] Knickmeyer RC, Gouttard S, Kang C, Evans D, Wilber K, et al (2008) A Structural MRI Study of Human Brain Development from Birth to 2 Years. Journal of Neuroscience 28: 12176–12182. 19020011 10.1523/JNEUROSCI.3479-08.2008 PMC2884385

[50] Nigg JT (2000) On inhibition/disinhibition in developmental psychopathology: views from cognitive and personality psychology and a working inhibition taxonomy. Psychological bulletin 126: 220–246. 10748641 10.1037/0033-2909.126.2.220

[51] Almli CR, Rivkin MJ, McKinstry RC (2007) The NIH MRI study of normal brain development (Objective-2): Newborns, infants, toddlers, and preschoolers. NeuroImage 35: 308–325. 17239623 10.1016/j.neuroimage.2006.08.058

[52] Liu J (2004) Childhood externalizing behavior: theory and implications. J Child Adolesc Psychiatr Nurs 17: 93–103. 15535385 10.1111/j.1744-6171.2004.tb00003.x PMC1617081

[53] Shattuck DW, Leahy RM (2001) Automated graph-based analysis and correction of cortical volume topology. IEEE Transactions on Medical Imaging 20: 1167–1177. 11700742 10.1109/42.963819

[54] Shi F, Wang L, Dai Y, Gilmore JH, Lin W, et al (2012) LABEL: Pediatric Brain Extraction using Learning-based Meta-algorithm. NeuroImage 62: 1975–1986. 22634859 10.1016/j.neuroimage.2012.05.042 PMC3408835

[55] Sled JG, Zijdenbos AP, Evans AC (1998) A nonparametric method for automatic correction of intensity nonuniformity in MRI data. IEEE Trans Med Imaging 17: 87–97. 9617910 10.1109/42.668698

[56] Ashburner J, Friston KJ (2005) Unified segmentation. NeuroImage 26: 839–851. 15955494 10.1016/j.neuroimage.2005.02.018

[57] Shi F, Yap P-T, Wu G, Jia H, Gilmore JH, et al (2011) Infant Brain Atlases from Neonates to 1- and 2-Year-Olds. PLoS ONE 6: e18746 doi:10.1371/journal.pone.0018746 21533194 10.1371/journal.pone.0018746 PMC3077403

[58] Shi F, Fan Y, Tang S, Gilmore JH, Lin W, et al (2010) Neonatal brain image segmentation in longitudinal MRI studies. NeuroImage 49: 391–400. 19660558 10.1016/j.neuroimage.2009.07.066 PMC2764995

[59] Tzourio-Mazoyer N, Landeau B, Papathanassiou D, Crivello F, Etard O, et al (2002) Automated anatomical labeling of activations in SPM using a macroscopic anatomical parcellation of the MNI MRI single-subject brain. NeuroImage 15: 273–289. 11771995 10.1006/nimg.2001.0978

[60] Macomber J, Pergamit M (2009) Vulnerable Youth and the Transition to Adulthood Available: http://aspe.hhs.gov/hsp/09/vulnerableyouth/4/index.pdf Accessed 22 July 2013.

[61] Case A, Lubotsky D, Paxson C (2002) Economic Status and Health in Childhood: The Origins of the Gradient. The American Economic Review 92: 1308–1334. 29058397 10.1257/000282802762024520

[62] Hackman DA, Farah MJ (2009) Socioeconomic status and the developing brain. Trends in Cognitive Sciences 13: 65–73. 19135405 10.1016/j.tics.2008.11.003 PMC3575682

[63] Blakemore SJ, Frith U (2005) The Learning Brain: Lessons for education. Malden: Blackwell Publishing. 216 p. 10.1111/j.1467-7687.2005.00434.x 16246234

[64] Honey CJ, Sporns O (2008) Dynamical consequences of lesions in cortical networks. Human brain mapping 29: 802–809. 18438885 10.1002/hbm.20579 PMC6870962

[65] Arnsten AFT (2009) Stress signalling pathways that impair prefrontal cortex structure and function. Nat Rev Neurosci 10: 410–422. 19455173 10.1038/nrn2648 PMC2907136

[66] Gianaros PJ, Horenstein JA, Cohen S, Matthews KA, Brown SM, et al (2007) Perigenual anterior cingulate morphology covaries with perceived social standing. Soc Cogn Affect Neurosci 2: 161–173. 18418472 10.1093/scan/nsm013 PMC2312334

[67] Hanson JL, Chandra A, Wolfe BL, Pollak SD (2011) Association between income and the hippocampus. PLoS ONE 6: e18712 doi:10.1371/journal.pone.0018712 21573231 10.1371/journal.pone.0018712 PMC3087752

[68] Raizada RD, Richards TL, Meltzoff A, Kuhl PK (2008) Socioeconomic status predicts hemispheric specialisation of the left inferior frontal gyrus in young children. NeuroImage 40: 1392–1401. 18308588 10.1016/j.neuroimage.2008.01.021 PMC2679945

[69] Sheridan MA, Fox NA, Zeanah CH, McLaughlin KA, Nelson CA (2012) Variation in neural development as a result of exposure to institutionalization early in childhood. Proc Natl Acad Sci U S A 109: 12927–12932. 22826224 10.1073/pnas.1200041109 PMC3420193

[70] Duncan GJ, Brooks-Gunn J, Klebanov PK (1994) Economic deprivation and early childhood development. Child Dev 65: 296–318. 7516849

[71] May A (2011) Experience-dependent structural plasticity in the adult human brain. Trends in Cognitive Sciences 15: 475–482. 21906988 10.1016/j.tics.2011.08.002

[72] Zatorre RJ, Fields RD, Johansen-Berg H (2012) Plasticity in gray and white: neuroimaging changes in brain structure during learning. Nature Neuroscience 15: 528–536. 22426254 10.1038/nn.3045 PMC3660656

[73] Chiang MC, McMahon KL, de Zubicaray GI, Martin NG, Hickie I, et al (2011) Genetics of white matter development: a DTI study of 705 twins and their siblings aged 12 to 29. NeuroImage 54: 2308–2317. 20950689 10.1016/j.neuroimage.2010.10.015 PMC3197836

