# Correction: Family Poverty Affects the Rate of Human Infant Brain Growth

**DOI:** 10.1371/journal.pone.0146434

**Published:** 2015-12-30

**Authors:** Jamie L. Hanson, Nicole Hair, Dinggang G. Shen, Feng Shi, John H. Gilmore, Barbara L. Wolfe, Seth D. Pollak

In Figs 2, 3 and 4 of the published article the units of volume are incorrectly noted as cm^3^. The correct units are mm^3^.

**Fig 2:**
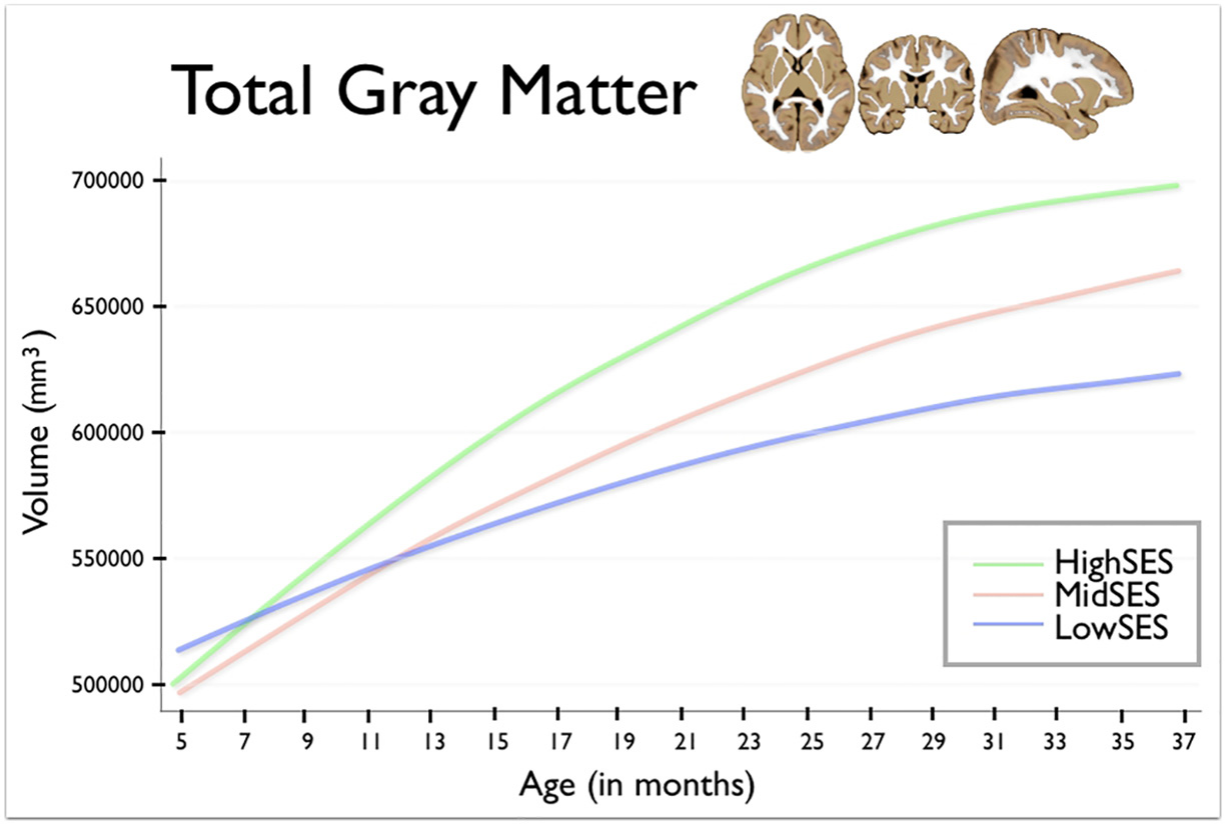
This figure shows total gray matter volume for group by age. Age in months is shown on the horizontal axis, spanning from 5 to 37 months. Total gray matter volume is shown on the vertical axis. The blue line shows children from Low SES households; children from Mid SES households are shown in red. The green line shows children from High SES households.

**Fig 3:**
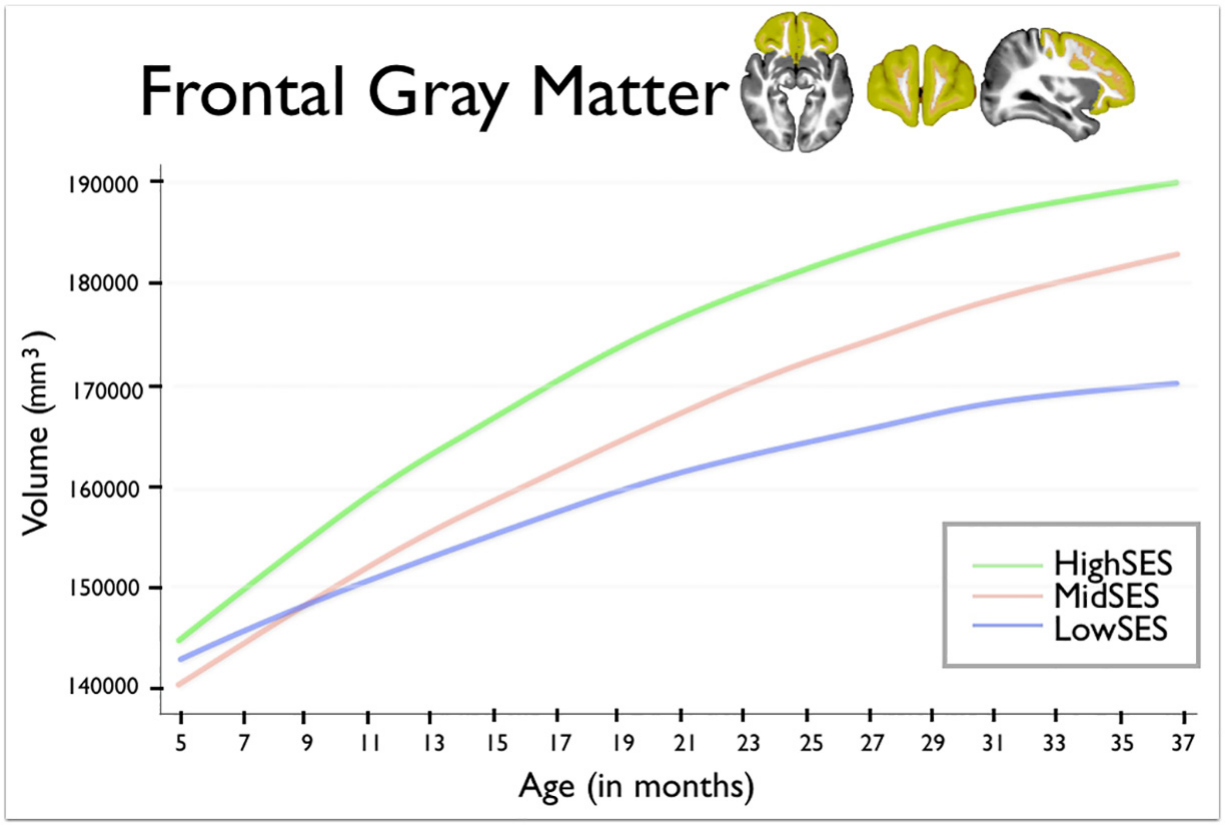
This figure shows frontal lobe gray matter volumes for group by age. Age in months is shown on the horizontal axis, spanning from 5 to 37 months. Total gray matter volume is shown on the vertical axis. The blue line shows children from Low SES households; children from Mid SES households are shown in red. The green line shows children from High SES households.

**Fig 4:**
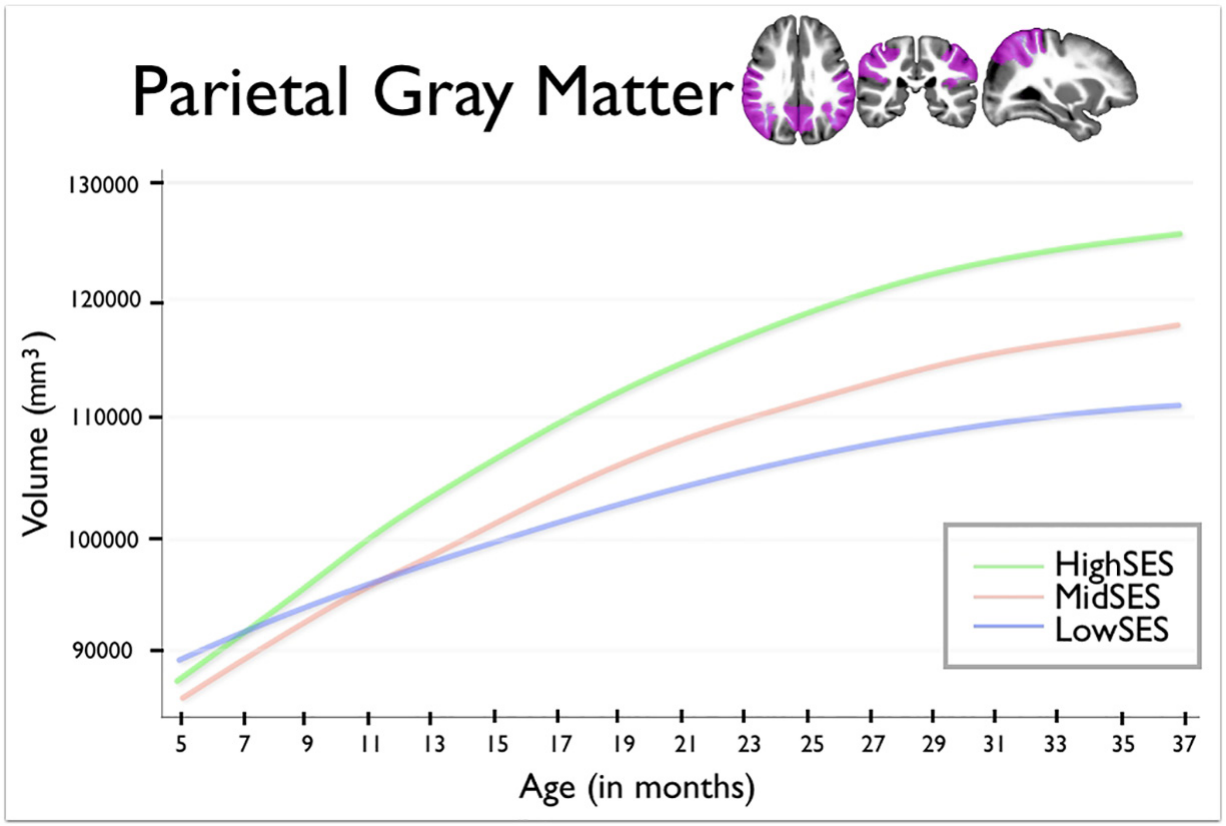
This figure shows parietal lobe gray matter volumes for group by age. Age in months is shown on the horizontal axis, spanning from 5 to 37 months. Total gray matter volume is shown on the vertical axis. The blue line shows children from Low SES households; children from Mid SES households are shown in red. The green line shows children from High SES households.
